# Inhomogeneity and Anisotropy in Nanostructured Melt-Spun Ti_2_NiCu Shape-Memory Ribbons

**DOI:** 10.3390/ma13204606

**Published:** 2020-10-16

**Authors:** Pranav Bhale, Pnina Ari-Gur, Victor Koledov, Alexander Shelyakov

**Affiliations:** 1 Department of Mechanical and Aerospace Engineering, Western Michigan University, Kalamazoo, MI 49008, USA; pranav.bhale@wmich.edu; 2 Kotelnikov Institute of Radioengineering and Electronics of RAS (Kotelnikov IRE RAS), 125009 Moscow, Russia; victor_koledov@mail.ru; 3 Department of Solid State Physics and Nanosystems, National Research Nuclear University “MEPhI” (Moscow Engineering Physics Institute), 115409 Moscow, Russia; alex-shel@mail.ru

**Keywords:** anisotropy, GIXD, inhomogeneity, melt-spinning, shape-memory, texture

## Abstract

Ti_2_NiCu exhibits outstanding properties, such as superelasticity. Recently, its functional properties were also demonstrated on the nanoscale, a fact that makes it the preferred choice for numerous applications. Its properties strongly depend on the manufacturing route. In this work, phase analysis, inhomogeneity, and texture of melt-spun Ti_2_NiCu ribbons were investigated using X-ray diffraction. Initially, the ribbons are amorphous. Passing an electric current result in controlled crystallization. Ribbons with 0%, 60%, and 96% crystallinity were studied. Both B2 austenite and B19 martensite phases were observed. Using grazing incidence X-ray diffraction, the inhomogeneity across the thickness was investigated and found to be substantial. At the free surface, a small presence of titanium dioxide may be present. Pole figures of 60% and 96% crystallinity revealed mostly strong fiber <100>_B2_ texture in the thickness direction. These observations may be inferred from the manufacturing route. This texture is beneficial. The inhomogeneity across the thickness has to be considered when designing devices.

## 1. Introduction

NiTiCu alloys demonstrate excellent properties, such as elastocaloric effect [1], shape-memory effect [2], and superelasticity [3]. Thanks to these properties, these alloys hold great promise for applications in multiple areas, such as bio-implants [4] and microelectromechanical systems (MEMS) [5]. At higher temperatures, the crystal structure of the austenitic phase in NiTi-based alloys is cubic (B2). Upon cooling, the B2 phase transforms into a martensitic phase that, depending on the composition, may have monoclinic, orthorhombic, or tetragonal crystal structure [6]. The reverse transformation of stress-induced martensite to austenite (superelasticity) is associated with a hysteresis under the stress–strain curve. Reversible superelastic strains of up to 13% [7] have been observed in some similar alloys, compared to only 0.2% in common metallic alloys. The addition of Cu reduces the hysteresis in NiTi alloy [8].

Some of the manufacturing routes used to obtain NiTi-based alloys include vacuum arc melting [9], powder metallurgy [10], and additive manufacturing [11]. To manufacture thin ribbons for applications like microactuators [12] and stents [13], the fast solidification melt-spinning process may also be used. In melt-spinning, hot molten alloy falls on a cold rotating drum, resulting in super-fast cooling, forming thin strips (“ribbons”) of the alloy. The ribbons produced by melt-spinning are usually 20–80 µm thick [14,15,16] and demonstrate inhomogeneity between the two sides of the ribbon; one is in contact with the cold drum while the other is exposed to ambient conditions [17]. As a result, these two sides and the thickness of the ribbon experience different cooling rates and environments, and these, in turn, affect the structure and properties of the alloy on each side, as well as across the thickness of the ribbon. The process also imparts a strong texture. For example, phases found in melt-spun ribbons of Ni_51_Ti_49_ alloy showed a strong presence of fiber texture along <100>_B2_ in the thickness direction of the ribbon, which increased the shape-memory effect and narrowed the phase transformation temperature range [18].

As the shape-memory and the superelastic properties of NiTiCu alloy critically depend on the crystalline structure present [19], it is important to study their nature and transformation. In this research, Ti_2_NiCu ribbons, melt-spun, and annealed (by electric pulses) were studied by X-ray diffraction to identify the phases, gradients of phases across the thickness, and the presence and nature of the preferred crystallographic orientation (a critical property to the ribbons performance [20]). In previous works [21,22], we have demonstrated their good two-way shape-memory behavior, which led to their application in nanotweezers, based on a composite of Ti_2_NiCu with a thin elastic layer of Pt deposited on it [21,22].

## 2. Materials and Methods

Amorphous Ti_2_NiCu ribbons were prepared by the melt-spinning technique. Molten alloy of high purity Ni, Ti, and Cu (MEPhI, Moscow, Russia) was poured on a fast-rotating copper wheel at a constant cooling rate of around 10^6^ K/s. The cooling rate in melt-spinning was estimated using the linear speed of the rotating drum (35 m/s) using the method described in reference [23]. This yielded ribbons that were 2 mm wide and 40 µm thick. The two sides of the ribbons experience different cooling rates; the air side is cooled by ambient air while the wheel side is cooled by the chilled drum. To obtain different degrees of crystallization in these ribbons, annealing was carried out by passing electric pulses through the ribbons for different durations, as previously described in detail [24]. The degree of average crystallinity (ϕ_r_) of ribbons was determined by Equation (1) [24], where R_a_ and R_c_ are the electrical resistance of completely amorphous and fully crystalline samples, respectively, and R_s_ is the resistance of the sample. For this research, ribbons with 0%, 60%, and 96% degrees of average crystallinity (ID R0, R60, and R96, respectively) were studied.

ϕ_r_ = (R_a_−R_s_)/(R_a_−R_c_) × 100%, (1)

Crystal structure phase analyses were conducted on each side (air and wheel) of each ribbon using X-ray diffraction (XRD) (Malvern-Panalytical Empyrean, Almelo, Overijssel, Netherlands) technique with scans from 20 to 100° 2θ.

Due to the nature of the melt-spinning process that imparts varying cooling rates and environments, the ribbons are expected to be inhomogeneous across their thickness. To study the inhomogeneity of crystalline structure across the depth, grazing incidence diffraction (GIXD) (Malvern-Panalytical Empyrean, Almelo, Overijssel, The Netherlands) technique was used to be able to control the X-ray beam penetration into the depth of the sample. This penetration depth (x) depends on the wavelength, the sample composition and density, and the linear absorption coefficient (µ) (in cm^−1^) of the sample under investigation and was calculated using Equation (2) [25]. The unitless constant K_x_ (2.30 [25] for this experiment) depends on the fraction of the total diffracted intensity from the surface of the sample up to a depth x (in µm). An incident angle (2ω), held constant throughout the experiment, was used to calculate the penetration depth. The theoretical density of the crystalline phase (6400 kg/m^3^) was used as the density of Ti_2_NiCu alloy.

x = [(K_x_·sin ω)/{μ·(1 + sin ω)}] × 10,000, (2)

To calculate µ for Ti_2_NiCu, mass attenuation coefficients of Ti (204 cm^2^/g), Ni (49.3 cm^2^/g), and Cu (52.7 cm^2^/g) were used [26]. For 99% of total diffracted intensities and calculated µ as 756.9 cm^−1^ (for Ti_2_NiCu alloy), penetration depths of 0.5, 1, and 2.65 µm at incident angles 2°, 4°, and 10°, respectively, were determined based on Equation (2). GIXD was done on each side of R0, R60, and R96 ribbons.

To study the crystallographic texture, pole figures of B19 (022), B19 (200), B19 (020), B2 {110}, and B2 {200} peaks were constructed to analyze the nature of the preferred orientation in the ribbons. Both sides of the 60 and 96% crystalline ribbons were measured in back reflection up to a tilt angles (χ) of 85°.

All 2θ-scans, GIXD, and pole-figure experiments were performed on a Malvern Panalytical Empyrean diffractometer using Cu Kα radiation (λ = 0.1541 nm).

## 3. Results and Discussion

### 3.1. X-ray Diffraction (XRD)

**Figure 1:**
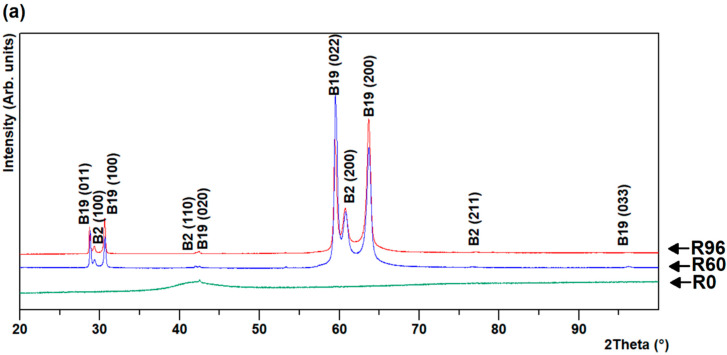

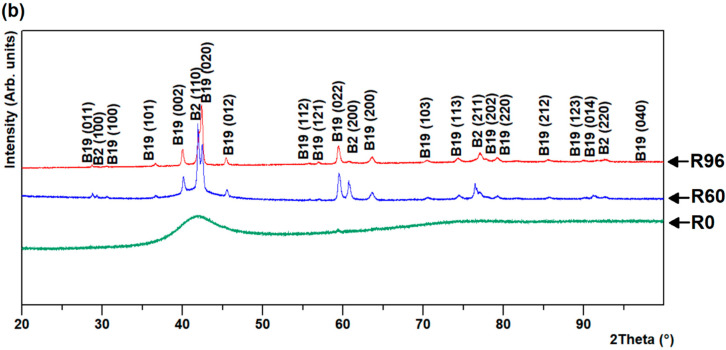
XRD scans of (**a**) air- and (**b**) wheel-side in R0, R60, and R96 ribbons.

Results of 2θ-scans at room temperature of melt-spun ribbons with 0, 60, and 96% approximate average crystallinity, confirmed our previous results [27] of a co-existence of cubic (B2) austenitic and orthorhombic (B19) martensitic phases on both the air- and wheel-side of R60 and R96 ribbons. The lattice parameter, calculated from the XRD scan peaks, were a = 0.305 nm (space group = 221 [27]) for the B2 phase and lattice parameters a = 0.292 nm, b = 0.428 nm, and c = 0.455 nm (space group = 51 [27]) for the B19 phase. The presence of a very broad peak near 40° (2θ) on both sides of the R0 ribbon may be attributed to the presence of the nanostructured phase, along the amorphous phase. Increasing heat-treatment times led to gradual crystallization first into the B2 phase, followed by a transition into the B19 phase in the R60 and R96 ribbons. The two sides of the R60 and R96 ribbons exhibited a substantial difference in the relative intensities between the B2 and B19 phases, whereas the R0 ribbon was almost identical on both sides, as shown in Figure 1a,b. The preferred orientation of the {110} and {100} peaks of the B2 austenite decreased on both sides (of R60 and R96 ribbons) with an increased degree of average crystallinity from 60 to 96%.

### 3.2. Grazing Incidence Diffraction (GIXD)

**Figure 2:**
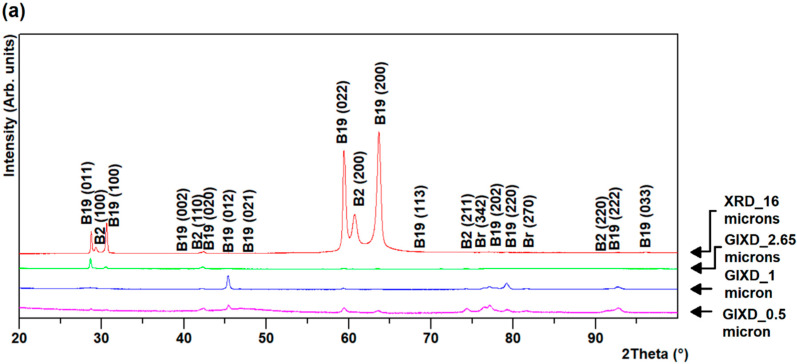

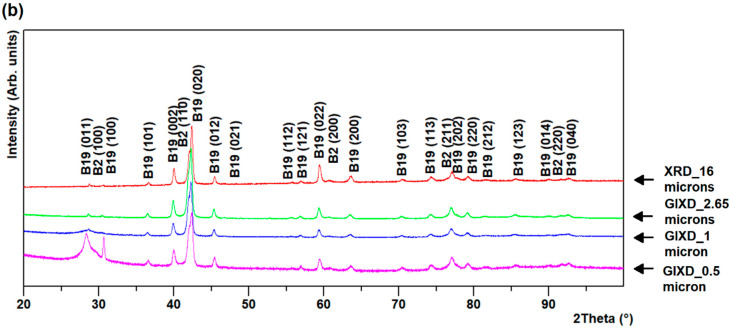
Grazing incidence diffraction (GIXD) for R96 ribbon (**a**) air- and (**b**) wheel-side.

**Figure 3:**
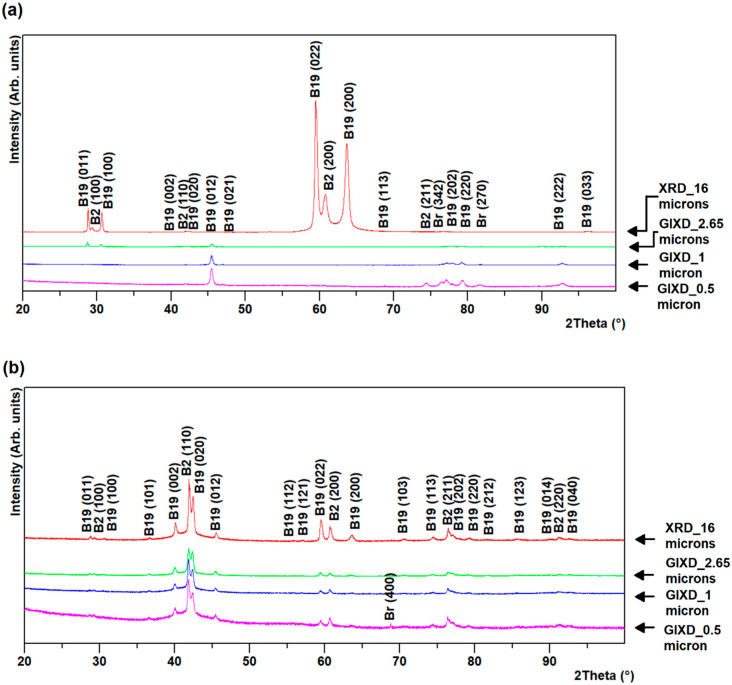
GIXD for R60 ribbon (**a**) air- and (**b**) wheel-side.

**Figure 4:**
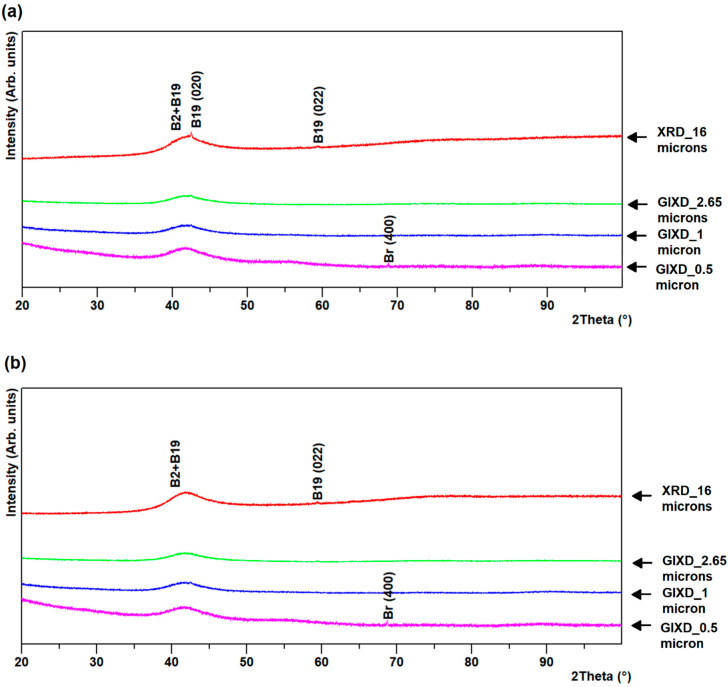
GIXD for R0 ribbon (**a**) air- and (**b**) wheel-side.

GIXD of R60 and R96 ribbons revealed a variation of the phase fractions across the thickness, whereas GIXD of R0 ribbons revealed almost uniform results throughout the thickness, in spite of the significantly different cooling rates between the surfaces and the core of the ribbon. At shallow depth (about 0.5 µm), very near the ribbon surface, a very small presence of a couple of extra peaks seems to suggest the presence of titanium dioxide (TiO_2_), brookite. This assumption is based on their presence only close to the free surface and their match with expected brookite peaks. This oxide was present on the air-side of the R96 ribbon and on both sides of R0 and R60 ribbons, as observed from Figure 2, Figure 3 and Figure 4.

The broad peak at the background observed in the GIXD patterns of both sides of R60 and R96 ribbons results from the higher fraction of the amorphous phase present near the surface than at a depth closer to the center. This is expected due to the much higher cooling rates on the outside surfaces, compared to that at the layers under the surface, across the thickness of the ribbons. GIXD at 0.5 and 1 µm on the air-side of R60 and R96 ribbons revealed the change in preferred orientation as the vanishing of B2 austenite {100} and B19 martensite (011) peaks (at ~30° and ~60° 2θ) and the emergence of B19 martensite (012) peak at ~45° 2θ. The relative intensities of phases on the wheel-side of the ribbons were almost uniform throughout the layers compared to that on the air-side of the ribbons, as observed in Figure 2, Figure 3 and Figure 4. This may be attributed to less grain growth for layers on the wheel-side compared to those on the air-side of the ribbons.

### 3.3. Crystallographic Preferred Orientation

**Figure 5:**
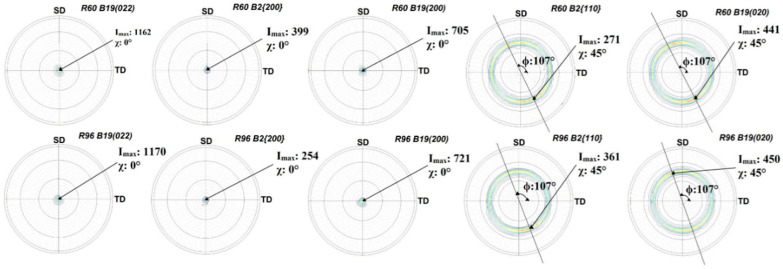
Air-side pole figures for R60 and R96 ribbons with spinning (SD) and transverse directions (TD) as reference.

**Figure 6:**
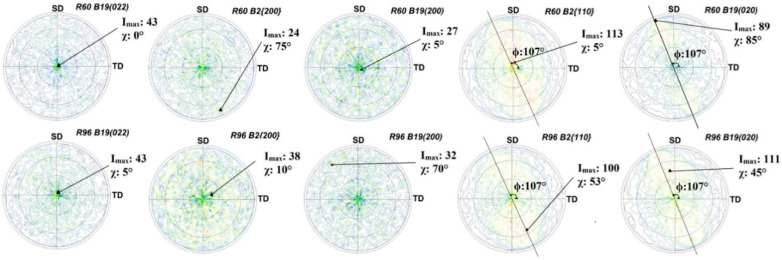
Wheel-side pole figures of R60 and R96 ribbons with spinning (SD) and transverse directions (TD) as reference.

The crystallographic texture on both sides of R60 and R96 is presented here as pole figures with spinning (SD) and transverse (TD) directions as sample reference coordinates. The air-side of these ribbons had a stronger texture compared to the wheel-side, as observed in Figure 5 and Figure 6. The maximum intensities of these pole figures with their respective tilt (χ) and rotation (φ) angles are summarized in Table 1. On the air-side of both ribbons, fiber texture, parallel to the thickness of the ribbon, was observed from B2 {200}, B19 (022), and B19 (200) pole figures. The pole figures of B2 {110} and B19 (020) planes confirmed the presence of the fiber texture but also revealed some presence of sheet texture (at χ~45°, φ~17° to SD).

The presence of texture, mostly in the air-side of the ribbons, is not surprising. The cooling rate of the surface in contact with air is substantially lower than the one in contact with the cold copper wheel. As a result, the air-side is partially crystalline. Solidification of the ribbon starts at the surface in contact with the cold wheel and progresses through the thickness to the air-side, giving rise to the development of fiber texture with the fiber axis parallel to the thickness of the ribbon.

The wheel-side of the R60 and R96 ribbons had insignificant preferred orientation, as observed in Figure 6. Cubic B2 {110} and orthorhombic B19 (020) pole figures show maximum intensity at a rotation angle ~17° counterclockwise to SD, which might be due to the angle made by these ribbons while coming out of the drum.

**Figure 7:**
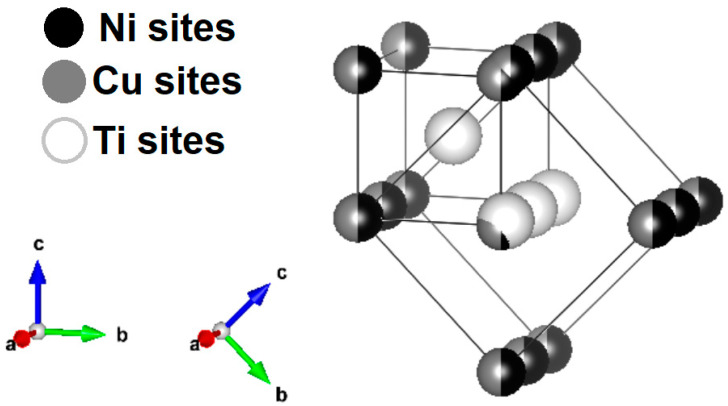
B2 and B19 phase crystal structures.

The crystallographic relationships between these phases (Equation (3)) were determined from the pole figures and were found to be similar to the well-known relationship. The B19 phase is rotated by 45° about the a-axis of the B2 phase. The [100], [011], and $\left[10\bar{1}\right]$ directions of the B2 phase then became the principal directions of the B19 phase, as shown in Figure 7.

B2 [01 ®1] || B19 [010]; B2 [011] || B19 [001]; B2 [100] || B19 [100](3)

It is interesting to note that the crystallographic texture present in R60 and R96 ribbons was very similar to that observed in pole figures of Ti_50_Ni_25_Cu_25_ melt-spun ribbons that were heat-treated in a furnace [28], and not by the passing pulses of electric current like the ribbons in the current study. This implies that the texture developed in heat-treated (annealed) ribbons is independent of the heat treatment route. It is, on the other hand, different than the [211] fiber texture found in a rolled sheet of Ti-Ni-Cu based alloy [29]. The presence of a weak sheet texture in these ribbons may be attributed to the shear parallel to the surface, resulting from the flow of semi-solid Ti_2_NiCu alloy over the already solidified layer in contact with the cold drum. The presence of <100> and <110> fiber textures of the parent phase in the air-side has been known to improve the shape-memory effect in NiTi-based alloys [18,30] and is desired in our ribbons as well, as our previous shape-memory results [21,22] demonstrate.

## 4. Conclusions

The processing of the Ti_2_NiCu ribbons resulted in a nanocrystalline, strongly textured, and inhomogeneous structure. The strong fiber <100>_B2_ texture parallel to the thickness direction and the associated B19 fiber texture substantially enhance the shape-memory properties and appear to stem mostly from the melt-spinning process. The pronounced inhomogeneity across the thickness needs to be considered in applications when the ribbon is etched or bonded on one side to another layer.

Recently the Ti_2_NiCu alloy-based composites, have demonstrated the outstanding functional properties on nanoscale [21]. The bottom-up nanointegration and nanomanipulation by the mechanical nanotools developed from Ti_2_NiCu melt-spun ribbons were developed [31]. The fine peculiarities of the structural disorder and texture properties found in the present work should be taken into account when developing the technology of mass production of the advanced mechanical nanodevices based on the amorphous crystalline melt-spun ribbons of the alloys with shape-memory effect.

## Figures and Tables

**Table 1:** Pole figure maximum intensities for R60 and R96 ribbons.

Side of Ribbon	Plane	Bragg Angle (2θ)	Tilt Angle (χ)	Rotation Angle (φ)	Maximum Intensity (Imax)	
					R60	R96
Air Side	B19 (022)	59.4°	0°	-	1162	1170
	B2 {200}	60.7°	0°	-	399	254
	B19 (200)	63.7°	0°	-	705	721
	B2 {110}	42.0°	45°	17°	271	361
	B19 (020)	42.4°	45°	17°	441	450
Wheel Side	B19 (022)	59.4°	0°	-	43	43
	B2 {200}	60.7°	0°	-	24	38
	B19 (200)	63.7°	0°	-	27	32
	B2 {110}	42.0°	45°	17°	113	100
	B19 (020)	42.4°	45°	17°	89	111
